# Decreased Dicer Expression Enhances SRP-Mediated Protein Targeting

**DOI:** 10.1371/journal.pone.0056950

**Published:** 2013-02-28

**Authors:** Yong-Feng Ren, Guiling Li, Yong-Feng Xue, Xue-Jiao Zhang, Yi-Jiang Song, Lu Lv, Jianmin Wu, Yu-Xiao Fang, Yu-Qun Wang, Ke-Qing Shi, Yong-ping Chen, Kai-Fu Tang

**Affiliations:** 1 Institute of Genomic Medicine, Wenzhou Medical College, Wenzhou, Zhejiang Province, People's Republic of China; 2 Department of Infection and Liver Diseases, Liver Research Center, Wenzhou Medical College, Wenzhou, Zhejiang Province, People's Republic of China; Institute of Molecular Genetics IMG-CNR, Italy

## Abstract

We have shown that Dicer processes 7SL RNA into different fragments ranging from ∼20 to more than 200 nucleotides. Here we addressed the molecular functions of these 7SL RNA fragments and found that some of them functioned as dominant-negative regulators of the full-length 7SL RNA, interfering with signal recognition particle (SRP) complex formation. Transfection of these 7SL RNA fragments inhibited the expression of cell surface glycoproteins, the targeting of a reporter protein to the endoplasmic reticulum, and the secretion of secreted alkaline phosphatase. These results suggest that some Dicer-processed 7SL RNA fragments interfered with SRP-mediated protein targeting. Moreover, we showed that Dicer knockdown enhanced SRP-mediated protein targeting and that transfection of a mixture of the 7SL RNA fragments partially restored this effect. Our data indicate that Dicer can fine-tune the efficiency of SRP-mediated protein targeting via processing a proportion of 7SL RNA into fragments of different lengths.

## Introduction

The signal recognition particle (SRP) is a key component of the cellular machinery that couples the ongoing synthesis of proteins to their proper subcellular compartments and is essential for cotranslational insertion of membrane and secretory proteins into the lumen of the endoplasmic reticulum (ER) [1], [2]. The cotranslational protein targeting process is characterized by a well-understood sequence of events as follows: The N-terminal signal sequence of a nascent polypeptide is recognized on the ribosome by SRP, and this SRP-ribosome complex is then targeted to the membrane via the SRP receptor. Finally, the nascent chain is transferred from SRP to the protein-conducting channel, through which it is cotranslationally threaded [3].

The eukaryotic SRP complex comprises six distinct polypeptides (SRP9, SRP14, SRP19, SRP54, SRP68, and SRP72) bound to an RNA molecule (the 7SL RNA) [4], [5]. Down-regulation of 7SL RNA leads to inefficient targeting of nascent polypeptides to the ER. Misra and colleagues reported that in macrophage-like cell lines (J774G8 and U937), targeting of proteins to the ER and plasma membrane and the secretion of proteins are compromised by infection with Leishmania due to down-regulation of 7SL RNA [6]. Knockdown of SRP14, SRP54, or SRP72 led to reduced levels of 7SL RNA and resulted in inefficient SRP-mediated protein targeting [7].

7SL RNA was first detected in Rous sarcoma virus particles [8] and later identified as a stable component of the SRP complex [4], [5]. 7SL RNA is packaged into HIV-1 virions, and an endoribonucleolytic fragment of 7SL RNA (termed 7SLrem) is present in HIV-1 virions and minimal virus-like particles [9], [10], [11], [12], [13]. The ends of 7SLrem map to bulges in the secondary structure of the full-length 7SL RNA where nucleotides remain unpaired, implying that the full-length 7SL RNA is processed into 7SLrem by a single-stranded endonuclease [10]. Recently, we found that Dicer, a double-stranded endonuclease involved in the biogenesis of microRNA (miRNA) and small interfering RNA (siRNA) [14], [15], also processes 7SL RNA into fragments ranging from ∼20 to more than 200 nucleotides (nt) [16]. We also showed that the ∼20 nt RNAs did not function like miRNAs, nor did they regulate the expression of 7SL RNA [16].

In the present study, we investigated the cellular functions of the Dicer-processed 7SL RNA fragments and found that some function as dominant-negative regulators of the full-length 7SL RNA by interfering with the formation of the SRP complex and inhibiting SRP-mediated protein targeting.

## Materials and Methods

### Cell Culture, siRNAs, Plasmids and Transfection

HepG2.2.15 and HEK293T cells were cultured in RPMI 1640 medium (Hyclone, Logan, UT, USA), RKO and T29 cells were cultured in DMEM (Hyclone), and HCT116 cells were cultured in McCoy's 5A medium (Gibco, GrandIsland, NY, USA). All media were supplemented with 10% fetal bovine serum. We obtained siRNAs from Invitrogen (Life Technologies, Shanghai, China) and constructed plasmids expressing shRNAs (shDCR and shCon) by inserting the corresponding hairpin oligonucleotides into the pSUPER.neo+GFP vector (Oligoengine, Seattle, WA, USA). The target sequences of siRNAs and shRNAs were as follows: Dicer (AAGGCTTACCTTCTCCAGGCT), Control (AATTCTCCGAACGTGTCACGT). RNA transfection was performed as described [17], [18], [19]. The efficiency of interference was validated using either real-time RT-PCR or western blotting, or both. Plasmid transfection was performed using Lipofectamine 2000 (Invitrogen, Grand Island, NY, USA) according to the manufacturer's instructions.

### RNA Preparation

The control RNA (a fragment of LacZ) was previously described [16]. The 7SL RNA fragments (including 7SL(1-96), 7SL(1-212), and 7SL(97-299)) were transcribed *in vitro* according to a published method [20] and gel-purified using PAGE under denaturing conditions (7 M urea). The templates used for *in vitro* transcription were PCR-amplified from the pMD-7SL plasmid [16] using the following primers: 7SL(1-96), 5′-AAGCTTAATTATAATACGACTCACTATAG-3′ and 5′-TAGCGCACTACAGCCCAGAACTC-3′; 7SL(1-212), 5′-AAGCTTAATTATAATACGACTCACTATAG-3′ and 5′-GACCTGCTCCGTTTCCGACCTGG-3′; 7SL(97-299), 5′-AATTATAATACGACTCACTATAGGGAGACCGCGCCCGGCCTGATGAGTCCGTGAGGACGAAACGGTACCCGGTACCGTCTGCCGATCGGGTGTCCGCAC-3′ and 5′-AGAGACGGGGTCTCGCTATGTTG-3′.

### Western Blotting

Cells were lysed in RIPA buffer and equal amounts of denatured protein were subjected to SDS-PAGE and transferred to polyvinylidene fluoride membranes (Millipore, Bedford, MA, USA). Membranes were then incubated with primary antibody, followed by horseradish peroxidase-linked secondary antibody, and immune complexes were detected using ECL plus reagent (Millipore). The primary antibodies were as follows: Dicer and SRP19 (Abcam, Cambridge, MA, USA), SRP9 and SRP14 (Santa Cruz Biotechnology Inc., Santa Cruz, CA, USA), SRP54 and SRP68 (Proteintech, Chicago, IL, USA), SRP72 (Novus, Littleton, CO, USA), γ-tubulin and β-actin (Boster, Wuhan, China).

### Quantitative Real-time RT-PCR

Total RNA was prepared using TRIzol® reagent (Invitrogen) and incubated with RNase-free DNase I (Fermentas, Glen Burnie, MD, USA) for 30 min. The DNA-free RNA was reverse transcribed using a M-MLV reverse transcription kit (Promega, Madison, WI, USA) according to the manufacturer's instructions. Samples prepared in the absence of reverse transcriptase served as negative controls. SYBR green real-time PCR was performed using the ABI PRISM 7300 Sequence Detection system (Applied Biosystems). The level of *GAPDH* mRNA served as an internal control. Primer sequences used to detect Dicer and GAPDH mRNAs were as described [17]. Primer sequences (forward and reverse) for SRP proteins were as follows: SRP9, 5′-GGCTTCCTTGGCTACCATAA-3′ and GGCATTCTGATGGGAACTTG; SRP14, 5′-CAGCCCATCCATGTTAGCTC-3′ and AGCTACCGATGGGAAGAAGA-3′; SRP19, 5′-AGACTTTGGTCAGCACCTCC-3′ and AAACAGGAAGATGGGAGCCT-3′; SRP54, 5′-TTCCAAGGTCTGCTAGAACCA-3′ and CTCCCAGCTTTCTTGGGTTT-3′; SRP68, 5′-CGCTCCTTAGTTTCTTCGCTT-3′ and AGAGGGAGAACGGTTCCAGT-3′; SRP72, 5′-AGCCATCTTTCTGGATCTGG-3′ and TACATTCGGAAGAAGGGTGG-3′.

### Fractionation of Postnuclear Extracts to Analyze SRP Assembly

Fractionation of postnuclear extracts was carried out as described with modifications [21]. The HepG2.2.15 cells were homogenized in lysis buffer (50 mM Tris-HCl, pH 7.5, 0.25 M sucrose, 25 mM KCl, 5 mM MgCl_2_, 0.01% octaethylene glycol dodecyl ether (Nikko, Tokyo, Japan), 1 mM dithiothreitol, 30 U/ml RNase inhibitors (Promega), 0.25% Triton X-100, and a cocktail of protease inhibitors). The homogenized cells were incubated at 0°C for 10 min, potassium acetate was added to 500 mM, and nuclei were isolated by centrifuging the homogenate at 800×*g* for 10 min. The postnuclear fraction (1 mg) was incubated with 30 pmol 7SL RNA fragments or control LacZ RNA at 0°C for 10 min, 37°C for 60 min, layered onto a 10–40% glycerol gradient, and centrifuged at 5°C for 8 h at 40,000 rpm in a Beckman SW41Ti rotor. Ten equal fractions were collected from the top to the bottom of the gradient. One-third of each fraction was used for RNA extraction using TRIzol LS (Invitrogen), and the level of 7SL RNA was measured by real-time RT-PCR using the following primers, 5′-GGAGTTCTGGGCTGTAGTGC-3′ and 5′-ATCAGCACGGGAGTTTTGAC-3′ to specifically detect the full-length 7SL RNA. The remainder of each fraction was treated with trichloroacetic acid to precipitate proteins for western blot analysis.

### Detection of Cell Surface Glycoproteins

Cells were washed three-times with phosphate-buffered saline (PBS) and stained with Alexa Fluor® 488 conjugate-lectin GS-II (100 µg/ml, Invitrogen) at room temperature for 30 min. The cells were then washed three-times and resuspended in PBS. Fluorescence was detected using FACSAria (BD Biosciences) and analyzed using WinMDI software (http://facs.scripps.edu/software.html).

### ECFP-ER Expression

HEK293T cells were transfected with the plasmid pECFP-ER (Invitrogen). This plasmid encodes enhanced cyan fluorescent protein (ECFP) fused at its 5′ and 3′ ends to the endoplasmic reticulum (ER) targeting sequence of calreticulin and the ER retrieval sequence, KDEL, respectively. A cell line stably expressing pECFP-ER was transfected with specific RNAs as indicated, and images of fluorescent cells were obtained using a Leica DMI3000 B microscope. When compared with the corresponding control, the images were taken with the same light intensity. However, to present the results more clearly, different light intensities were applied in different experiments.

### Assays for Secreted Alkaline Phosphatase (SEAP)

Cells were co-transfected with a plasmid encoding SEAP (pSEAP2-control, BD Biosciences, San Jose, CA, USA) and different RNAs or shRNAs as indicated. The growth medium was changed every 24 h and collected at indicated time points. SEAP activity was determined using the Great EscAPe SEAP kit (BD Biosciences) following the manufacturer's instructions.

### Cloning of the Dicer-processed 7SL RNA Fragments

Small RNAs less than 200 nt were extracted from HepG2.2.15 cells using the E.Z.N.A. Micro RNA Kit (Omega, Norcross, GA, USA) and polyadenylated using the Poly(A) Polymerase Tailing Kit (Epicentre, Madison, WI). Reverse transcription was then performed using the SMARTScribe M-MLV Reverse Transcriptase System (Clontech, Mountain View, CA, USA) with CDS III/3′ PCR Primer and SMART IV Oligonucleotide. Next, the 7SL RNA derived-cDNA was annealed with a biotin-labeled 7SL sRNA5cd oligonucleotide (biotin-GAGTTCTGGGCTGTAGTGCGCTA) and purified using Dynabeads® M-280 Streptavidin magnetic beads (Invitrogen) according to the manufacturer's instructions. The purified DNA was PCR-amplified using CDS III/3′ PCR and 5′ PCR primers and cloned into the pGEM-T Easy Vector (Promega). Primer and oligonucleotide sequences are as follows:

CDS III/3′ PCR Primer (5′-ATTCTAGAGGCCGAGGCGGCCGACATGTTTTTTTTTTTTTTTTTTTTTTTTTTTTTTVN-3′); SMART IV Oligonucleotide (5′-AAGCAGTGGTATCAACGCAGAGTGGCCATTACGGCCGGG-3′); and 5′ PCR Primer (5′-AAGCAGTGGTATCAACGCAGAGT-3′).

### Statistical Analysis

Each experiment was independently performed at least three times. All data are shown as mean ± SD except as specifically indicated. Statistical significance was determined by Student's 2-tailed *t*-test, and results were considered significant at p<0.05 when compared with the control.

## Results

### The 7SL RNA-Derived sRNA5cd does not Modulate SRP-Mediated Protein Targeting

7SL sRNA5cd accounts for more than 95% of the ∼20 nt 7SL RNA-derived small RNAs [16]. To address whether Dicer-processed 7SL RNA fragments modulate the efficiency of SRP-mediated protein targeting, we first investigated the effect of 7SL sRNA5cd on the secretion of SEAP [22]. Co-transfection of the pSEAP2-control plasmid and different concentrations of 7SL sRNA5cd indicated that 7SL sRNA5cd did not significantly affect the secretion of SEAP (Fig. S1A). We then monitored the expression of a specific reporter protein ECFP-ER, which is an enhanced cyan fluorescent protein fused to the ER-targeting sequence of calreticulin. The fluorescence intensities in 7SL sRNA5cd-transfected cells were comparable to those in control small RNA-transfected cells (Fig. S1B). Finally, we measured the expression of cell surface glycoproteins in cells transfected with 7SL sRNA5cd or control small RNA. Our results indicated that transfection of 7SL sRNA5cd did not alter the expression of total cell surface glycoproteins (Fig. S1C). Taken together, these results indicate that 7SL sRNA5cd did not modulate the efficiency of SRP-mediated protein targeting.

### Long Dicer-Processed 7SL RNA Fragments Repress SRP-Mediated Protein Targeting and Interfere with Formation of the SRP Complex

**Figure 1:**
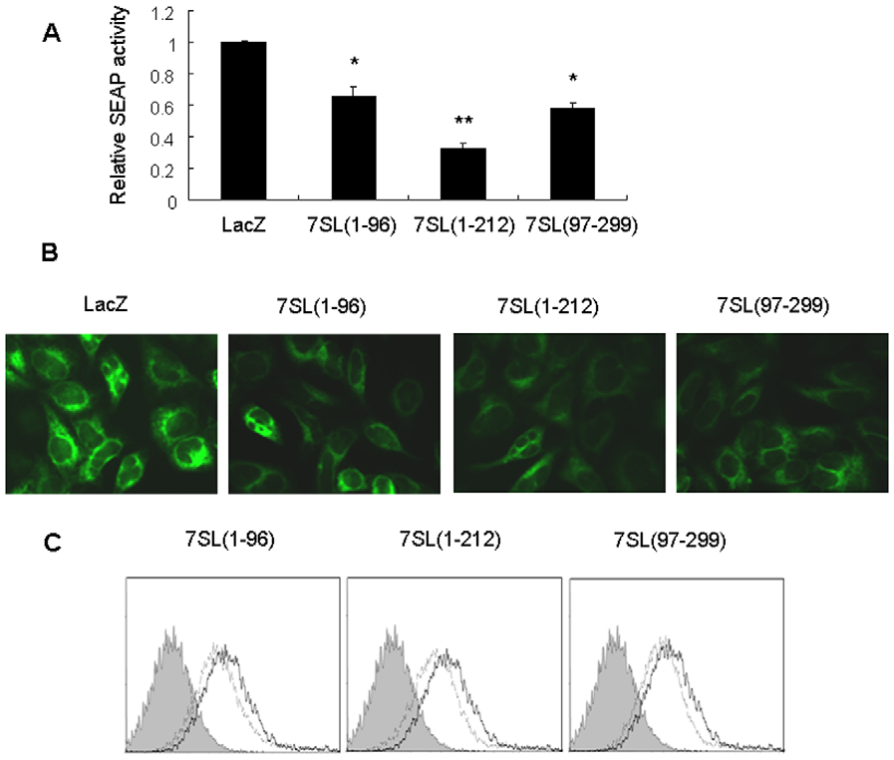
7SL RNA fragments repress SRP-mediated protein targeting. (A) HEK293T cells were co-transfected with the pSEAP2-control plasmid and different 7SL RNA fragments or the control RNA (LacZ RNA), and SEAP activity was determined 48 h post-transfection, *p<0.05, **p<0.01 compared with control RNA. (B) HEK293T cells stably expressing ECFP-ER were transfected with different 7SL RNA fragments or LacZ RNA. Fluorescence was detected 48 h after transfection. (C) HepG2.2.15 cells were transfected with different 7SL RNA fragments or LacZ RNA, and cell surface glycoproteins were measured 48 h after transfection. Horizontal and vertical axes denote intensity of fluorescence and number of events, respectively. The filled histogram represents unstained cells, the thick line represents cells transfected with LacZ RNA, and the dashed line represents cells transfected with the 7SL RNA fragment.

We reported that in addition to the ∼20 nt small RNAs (including 7SL sRNA5cd and 7SL sRNA8b), 7SL RNA is also processed by Dicer into longer fragments [16]. We developed a method to clone these long 7SL RNA fragments (Fig. S2). Sequence analysis of eight clones revealed that two contain an inserted sequence corresponding to nucleotides 1 to 96 of 7SL RNA. This result is consistent with our previous findings [16], which indicate that Dicer cleaves 7SL RNA at four major sites (Fig. S3A). To address whether these long Dicer-processed 7SL fragments can modulate SRP-mediated protein targeting, we synthesized three 7SL RNA fragments, including 7SL(1-96), 7SL(1-212), and 7SL(97-299) (Fig. S3B). Co-transfection experiment indicated that 7SL(1-96) inhibited SEAP secretion in a concentration-dependent manner (Fig. S4). Transfection of 7SL (1-96), 7SL(1-212) or 7SL(97-299) (80 nM each) significantly inhibited the secretion of SEAP (Figs. 1A and S5) as well as the expression of ECFP-ER and cell surface glycoproteins (Figs. 1B and 1C).

**Figure 2:**
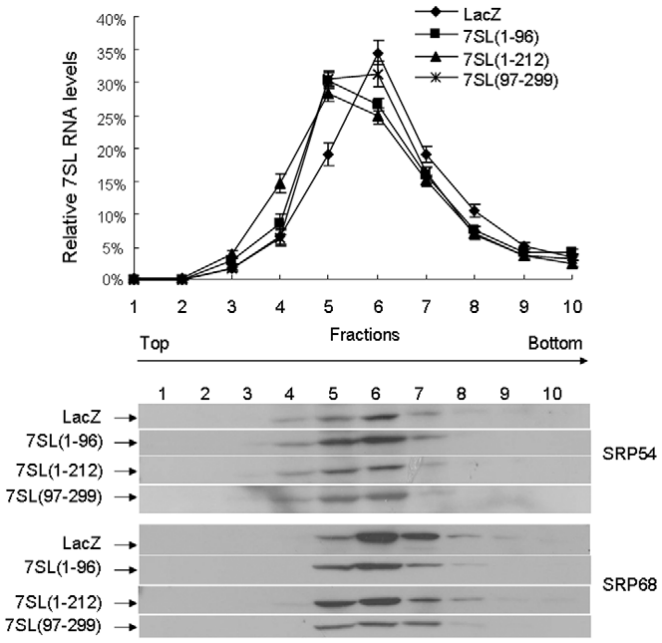
Effects of the 7SL RNA fragments on the formation of the SRP complex. Glycerol gradient fractionation of postnuclear extracts treated with either the 7SL RNA fragment or the control RNA (LacZ). Upper panel: The relative amount of 7SL RNA in each fraction. The sum of all fractions was set to 100%, and data are shown as mean ± SE from five independent experiments. Lower panel: representative image of western blotting analysis.

We then tested whether these 7SL RNA fragments interfere with the formation of the SRP complex. Postnuclear extracts incubated with the control RNA or 7SL RNA fragments were fractionated on glycerol gradients. As shown in Fig. 2, after incubation with the control RNA, most SRP54 and SRP68 proteins migrated in fractions 5 to 7 together with 7SL RNA. These SRP subunits and the full-length 7SL RNA shifted to the upper fractions after treatment with the synthetic 7SL RNA fragments, suggesting that part of the SRP complex may dissociate and hence migrate at lower sedimentation rates.

### Dicer Knockdown Enhances SRP-Mediated Protein Targeting

**Figure 3:**
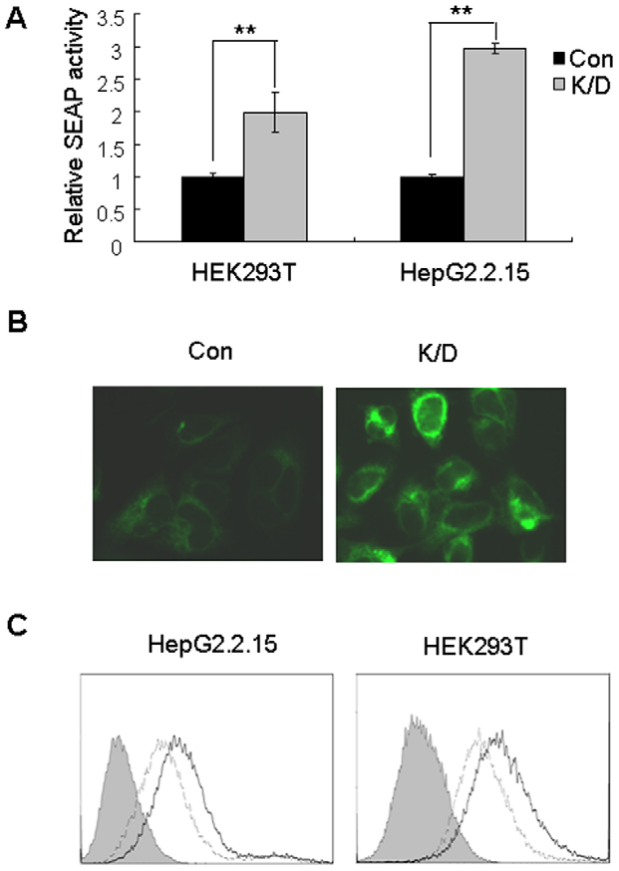
Dicer-knockdown enhances SRP-mediated protein targeting. (A) HepG2.2.15 and HEK293T cells were co-transfected with the SEAP-control and shDCR or shCon plasmids, and SEAP activity was determined 96 h later, **p<0.01 compared with the control. (B) HEK293T cells stably expressing with ECFR-ER were transfected twice with either siDCR or siCon. Fluorescence was detected 96 h after transfection. Con: control cells, K/D: Dicer knockdown cells. (C) HepG2.2.15 and HEK293T cells were transfected twice with siDCR or siCon, and cell surface glycoproteins were measured 96 h later. Horizontal and vertical axes denote intensity of fluorescence and the number of events, respectively. The filled histogram represents unstained cells, the thick line represents Dicer-knockdown cells, and dashed line represents the control cells.

We reported that Dicer-processed 7SL RNA fragments are reduced in Dicer-knockdown cells [16]. Here we show that some 7SL RNA fragments function as dominant-negative regulators of full-length 7SL RNA and inhibit SRP-mediated protein targeting (Figs. 1 and 2). Therefore, we hypothesized that Dicer knockdown may enhance SRP-mediated protein targeting. To test this hypothesis, we knocked-down Dicer expression in HepG2.2.15 and HEK293T cells (Fig. S6). Knockdown of Dicer significantly increased the secretion of SEAP (Fig. 3A) as well as the expression of ECFP-ER and cell surface glycoproteins (Fig. 3B and 3C). Similar results were obtained using additional cell lines (Fig. S6 and S7), which indicates that the effect of Dicer on SRP-mediated protein targeting is not cell-type specific.

**Figure 4:**
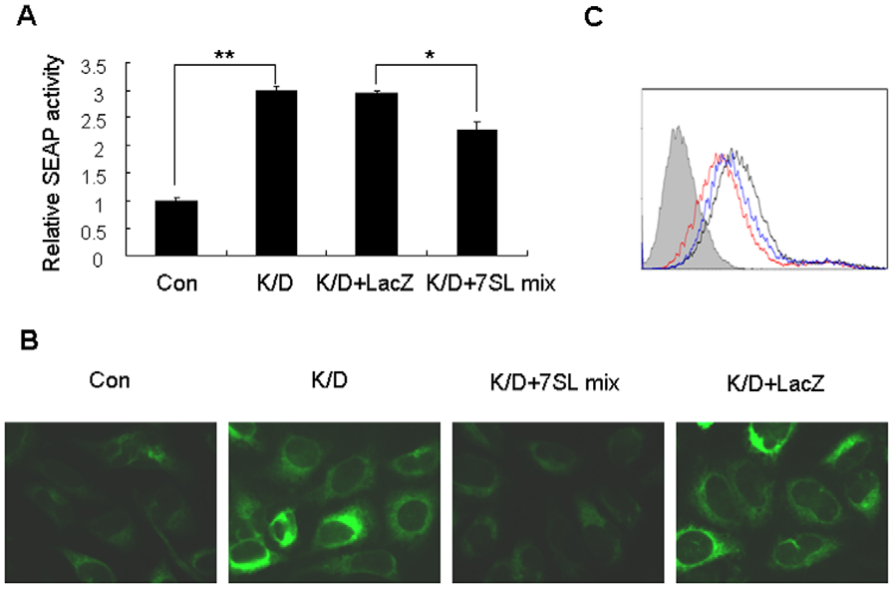
The 7SL RNA fragment mixture partially restores SRP function in Dicer-knockdown cells. (A) HepG2.2.15 cells were co-transfected for 48 h with the pSEAP2-control and either shDCR or shCon plasmids. The Dicer-knockdown cells (K/D) were then transfected with the 7SL RNA fragment mixture (7SL mix) or LacZ RNA, and SEAP activity was determined 48 h after the second transfection. *p<0.05, **p<0.01. (B) HEK293T cells that stably expressed ECFP-ER were pretreated with siDCR for 48 h, and then co-transfected with 7SL mix or LacZ RNA and siDCR. Fluorescence was detected 48 h after the second transfection. Cells treated twice with siDCR (K/D) or siCon (Con) served as controls. (C) HepG2.2.15 cells pretreated with siDCR for 48 h were co-transfected with 7SL mix and siDCR. The levels of cell surface glycoproteins were measured 48 h after the second transfection. Cells treated twice with siDCR or siCon served as controls. Horizontal and vertical axes denote intensity of fluorescence and the number of events, respectively. The filled histogram represents unstained cells, the red line represents cells transfected with siCon, the black line represents cells transfected with siDCR, and the blue line represents cells co-transfected with siDCR and the 7SL mix.

To further investigate whether enhanced SRP function in Dicer knockdown cells is due to reduction of Dicer-processed 7SL RNAs, we transfected Dicer-knockdown cells with a mixture of the 7SL RNA fragments, including 7SL(1-96), 7SL(1-212), and 7SL(97-299). The 7SL RNA fragment mixture not only partially inhibited the secretion of SEAP (Fig. 4A), but also partially restored the levels of ECFP-ER and cell surface glycoproteins in Dicer-knockdown cells (Fig. 4B and 4C). In contrast, transfection of Dicer-knockdown cells with LacZ RNA had no detectable effect (Fig. 4A, 4B, and data not shown).

Dicer is essential for the biogenesis of microRNAs that may target SRP subunits, thus decreased Dicer expression may result in elevated levels of SRP proteins. To exclude the possibility that the increased efficiency of ER-mediated protein targeting in Dicer knockdown cells was mediated by increased levels of SRP proteins, the expression of SRP subunits was monitored using real-time RT-PCR and western blotting analyses. We found that knockdown of Dicer did not alter the expression of SRP proteins (Fig. S8).

## Discussion

We previously demonstrated that Dicer processes 7SL RNA into fragments of different lengths [16]. In the present study, we demonstrate that several 7SL RNA fragments interfered with the formation of the SRP complex (Fig. 2), and thus inhibited the expression of ECFP-ER and cell surface glycoproteins as well as the secretion of SEAP (Fig. 1). Furthermore, knockdown of Dicer resulted in increased expression of ECFP-ER and cell surface glycoproteins and enhanced secretion of SEAP (Fig. 3), which were partially restored by transfection with the 7SL RNA fragment mixture (Fig. 4). Taken together, our data suggest that Dicer regulates SRP function via the following mechanism: Dicer processes a proportion of 7SL RNA into fragments of different lengths. Some of the 7SL RNA fragments function as dominant-negative regulators of the full-length 7SL RNA, interfering with the formation of the SRP complex and inhibiting its function. Decreased Dicer expression reduces the levels of the 7SL RNA fragments, thereby enhancing SRP-mediated protein targeting, which eventually increases the expression of cell surface glycoproteins and SEAP secretion.

Of note, 7SL RNA fragments longer than 200 nt or lacking the 7SL sRNA5cd sequence cannot be detected by our cloning method. Another limitation of this method is that any RNA shorter than 200 nt that contains a sequence similar to that of 7SL sRNA5cd can be cloned. Therefore, further effort is necessary to develop a more specific and efficient method to clone all of the 7SL RNA fragments.

## Conclusions

Our data indicate that Dicer can fine-tune the efficiency of SRP-mediated protein targeting via processing a proportion of 7SL RNA into fragments of different lengths that interfere with the function of the full-length 7SL RNA.

## Supporting Information

Figure S1 **7SL sRNA5cd does not modulate SRP-mediated protein targeting.** (A) HEK293T cells were co-transfected with the pSEAP2-control plasmid and different concentrations of 7SL sRNA5cd or the control small RNA. SEAP activity was determined 48 h post-transfection. (B) HEK293T cells stably expressing ECFR-ER were transfected with 7SL sRNA5cd or the control small RNA. Fluorescence was measured 48 h after transfection. (C) HepG2.2.15 cells were transfected with 7SL sRNA5cd or the control small RNA. Cell surface glycoproteins were measured 48 h later. Horizontal and vertical axes denote intensity of fluorescence and number of events, respectively. The filled histogram represents unstained cells, the black line represents cells transfected with the control small RNA, and the red line represents cells transfected with 7SL sRNA5cd.
(TIF)

Figure S2 **Flow chart for cloning long 7SL RNA fragments.** Small RNAs shorter than 200 nt were polyadenylated and reverse transcribed. The cDNAs derived from 7SL RNA fragments were purified using a biotin-labeled 7SL sRNA5cd oligonucleotide and PCR-amplified. The purified PCR products were cloned into the pGEM-T easy vector and sequenced.
(TIF)

Figure S3 **Dicer-processed 7SL RNA fragments.** (A) 7SL RNA Dicer cleavage sites (74, 96, 192, and 212) were predicated according to the sequences of 7SL sRNA5cd and 7SL sRNA8b. (B) Diagram of the synthetic Dicer-processed 7SL RNA fragments. Red arrows indicate the PCR primers that can only amplify the full-length 7SL RNA but not any of the synthetic 7SL RNA fragments
(TIF)

Figure S4 **7SL(1-96) inhibits secretion of SEAP in a concentration-dependent manner.** HEK293T cells were co-transfected with the pSEAP2-control plasmid and different amounts of 7SL(1-96) or LacZ RNA. SEAP activity was determined 48 h post-transfection, *p<0.05, **p<0.01 as compared with LacZ RNA.
(TIF)

Figure S5 **7SL(1-212) inhibits the secretion of SEAP in different human cell lines.** The pSEAP2-control plasmid was co-transfected with 7SL(1-212) or LacZ RNA, and SEAP activity was determined 48 h post transfection. **p<0.01 compared with LacZ RNA.
(TIF)

Figure S6 **Knockdown of Dicer in different human cell lines.** Representative western blot of Dicer. Detection of β-actin was used as the loading control. Con: control cells, K/D: Dicer knockdown cells.
(TIF)

Figure S7 **Dicer knockdown enhances the expression of cell surface glycoproteins.** Cells were transfected twice with siDCR or siCon, and cell surface glycoproteins were measured 96 h later. Horizontal and vertical axes denote intensity of fluorescence and number of events, respectively. The filled histogram represents unstained cells, the thick line represents Dicer knockdown cells, and the dashed line represents the control cells.
(TIF)

Figure S8 **Dicer knockdown does not alter the expression of SRP proteins.** HepG2.2.15 cells were transfected twice with siDCR or siCon, the expression of SRP proteins were measured by real-time RT-PCR (upper panel) and western blotting (lower panel) 96 h after transfection. Detection of β-actin was used as loading control. Con: control cells, K/D: Dicer knockdown cells.
(TIF)
