# Supplementary figures and images for: Decreased Dicer Expression Enhances SRP-Mediated Protein Targeting
Source: PLoS One. 2013 Feb 28;8(2):e56950. doi: 10.1371/journal.pone.0056950 (PMC3585229; doi:10.1371/journal.pone.0056950)

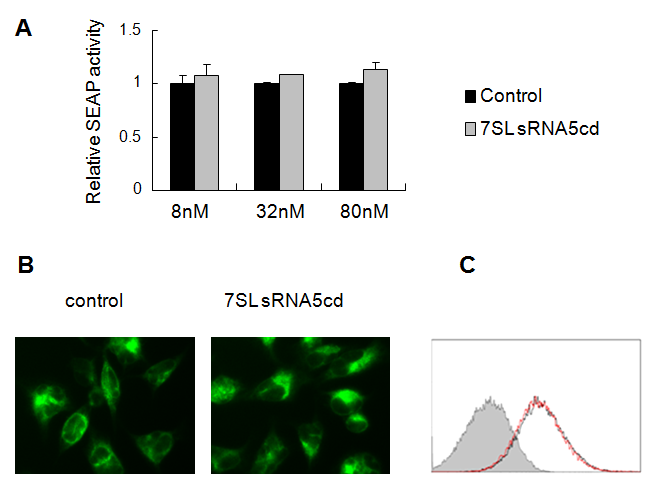

Supplement: Figure S1 — 7SL sRNA5cd does not modulate SRP-mediated protein targeting. (A) HEK293T cells were co-transfected with the pSEAP2-control plasmid and different concentrations of 7SL sRNA5cd or the control small RNA. SEAP activity was determined 48 h post-transfection. (B) HEK293T cells stably expressing ECFR-ER were transfected with 7SL sRNA5cd or the control small RNA. Fluorescence was measured 48 h after transfection. (C) HepG2.2.15 cells were transfected with 7SL sRNA5cd or the control small RNA. Cell surface glycoproteins were measured 48 h later. Horizontal and vertical axes denote intensity of fluorescence and number of events, respectively. The filled histogram represents unstained cells, the black line represents cells transfected with the control small RNA, and the red line represents cells transfected with 7SL sRNA5cd. (TIF) [file pone.0056950.s001.tif]

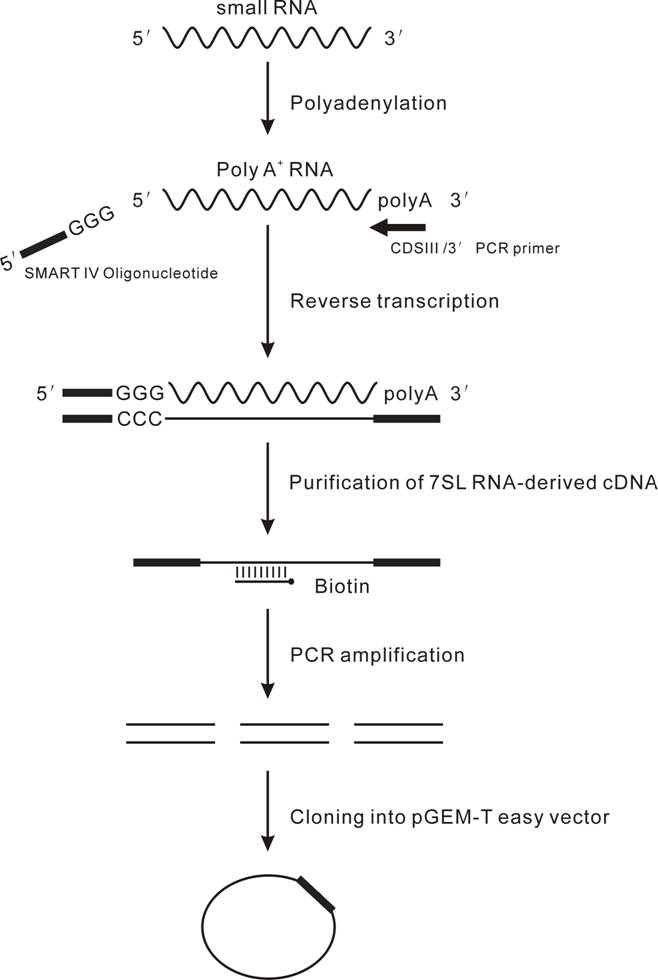

Supplement: Figure S2 — Flow chart for cloning long 7SL RNA fragments. Small RNAs shorter than 200 nt were polyadenylated and reverse transcribed. The cDNAs derived from 7SL RNA fragments were purified using a biotin-labeled 7SL sRNA5cd oligonucleotide and PCR-amplified. The purified PCR products were cloned into the pGEM-T easy vector and sequenced. (TIF) [file pone.0056950.s002.tif]

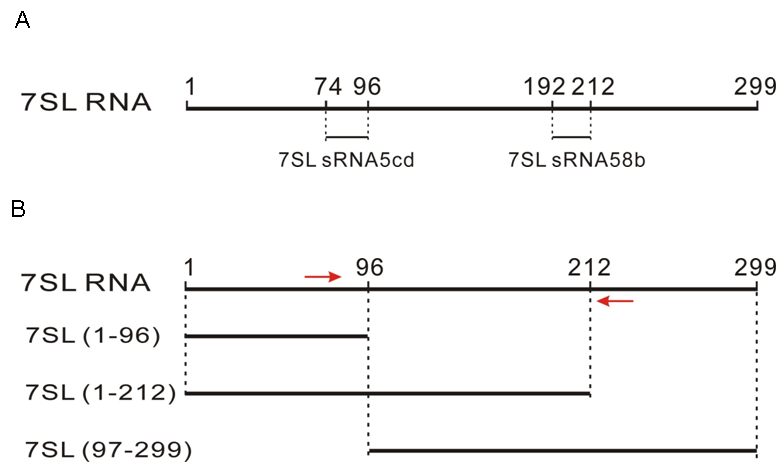

Supplement: Figure S3 — Dicer-processed 7SL RNA fragments. (A) 7SL RNA Dicer cleavage sites (74, 96, 192, and 212) were predicated according to the sequences of 7SL sRNA5cd and 7SL sRNA8b. (B) Diagram of the synthetic Dicer-processed 7SL RNA fragments. Red arrows indicate the PCR primers that can only amplify the full-length 7SL RNA but not any of the synthetic 7SL RNA fragments (TIF) [file pone.0056950.s003.tif]

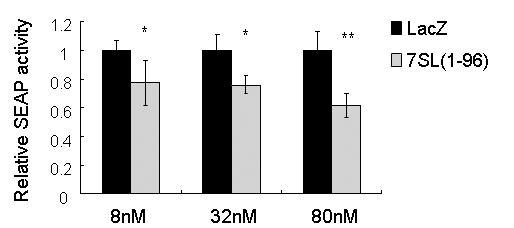

Supplement: Figure S4 — 7SL(1-96) inhibits secretion of SEAP in a concentration-dependent manner. HEK293T cells were co-transfected with the pSEAP2-control plasmid and different amounts of 7SL(1-96) or LacZ RNA. SEAP activity was determined 48 h post-transfection, *p<0.05, **p<0.01 as compared with LacZ RNA. (TIF) [file pone.0056950.s004.tif]

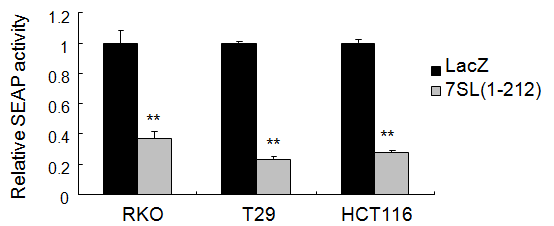

Supplement: Figure S5 — 7SL(1-212) inhibits the secretion of SEAP in different human cell lines. The pSEAP2-control plasmid was co-transfected with 7SL(1-212) or LacZ RNA, and SEAP activity was determined 48 h post transfection. **p<0.01 compared with LacZ RNA. (TIF) [file pone.0056950.s005.tif]

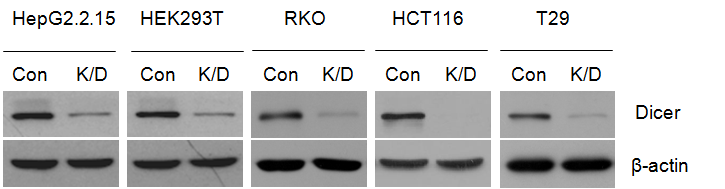

Supplement: Figure S6 — Knockdown of Dicer in different human cell lines. Representative western blot of Dicer. Detection of β-actin was used as the loading control. Con: control cells, K/D: Dicer knockdown cells. (TIF) [file pone.0056950.s006.tif]

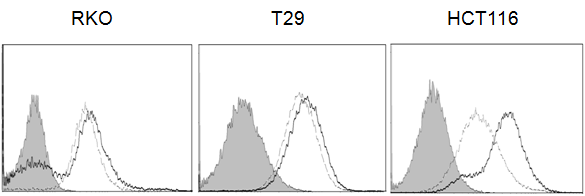

Supplement: Figure S7 — Dicer knockdown enhances the expression of cell surface glycoproteins. Cells were transfected twice with siDCR or siCon, and cell surface glycoproteins were measured 96 h later. Horizontal and vertical axes denote intensity of fluorescence and number of events, respectively. The filled histogram represents unstained cells, the thick line represents Dicer knockdown cells, and the dashed line represents the control cells. (TIF) [file pone.0056950.s007.tif]

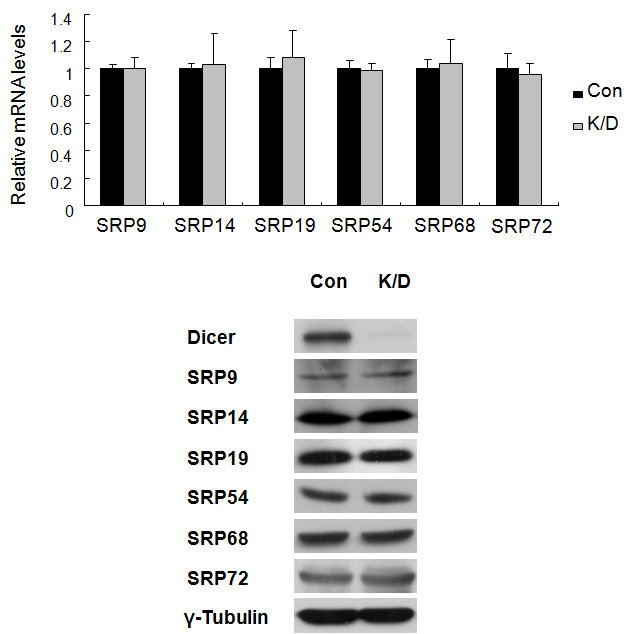

Supplement: Figure S8 — Dicer knockdown does not alter the expression of SRP proteins. HepG2.2.15 cells were transfected twice with siDCR or siCon, the expression of SRP proteins were measured by real-time RT-PCR (upper panel) and western blotting (lower panel) 96 h after transfection. Detection of β-actin was used as loading control. Con: control cells, K/D: Dicer knockdown cells. (TIF) [file pone.0056950.s008.tif]
